# Liver Transplantation for Unresectable Colorectal Liver Metastases: Evidence, Patient Selection, and Clinical Implementation

**DOI:** 10.3390/cancers18142306

**Published:** 2026-07-17

**Authors:** Tor Magnus Smedman, Åsmund Avdem Fretland, Svein Dueland, Jon Solheim, Morten Hagness, Kristoffer Lassen, Pål Dag Line, Sheraz Yaqub

**Affiliations:** 1 Transplant Oncology Research Group, Division of Surgery and Specialized Medicine, Oslo University Hospital, 0424 Oslo, Norway; t.m.smedman@medisin.uio.no (T.M.S.); svedue@ous-hf.no (S.D.); uxsojc@ous-hf.no (J.S.); mhagness@ous-hf.no (M.H.); p.d.line@medisin.uio.no (P.D.L.); 2 Department of Oncology, Oslo University Hospital, 0424 Oslo, Norway; 3 Department of HBP Surgery, Oslo University Hospital, 0424 Oslo, Norway; aafret@ous-hf.no (Å.A.F.); krlass@ous-hf.no (K.L.); 4 The Intervention Centre, Oslo University Hospital, 0424 Oslo, Norway; 5 Department of Transplantation Medicine, Oslo University Hospital, 0424 Oslo, Norway; 6 Institute of Clinical Medicine, University of Oslo, 0318 Oslo, Norway; 7 Department of Clinical Medicine, UiT the Arctic University of Norway, 9037 Tromsø, Norway

**Keywords:** colorectal cancer liver metastases, liver transplantation, liver surgery, transplant oncology

## Abstract

Liver transplantation (LT) has re-emerged as a potentially curative treatment for carefully selected patients with unresectable, liver-only colorectal liver metastases (CRLM). Prospective trials (SECA-I/II) demonstrated 5-year overall survival (OS) rates of 60–83% in selected patients, findings recently corroborated by the randomized TransMet trial, which showed superior 5-year OS with LT compared with chemotherapy alone. Favorable outcomes depend on rigorous patient selection integrating clinical risk stratification (Oslo Score), tumor burden assessed by metabolic tumor volume on FDG-PET-CT, and molecular profiling, while excluding high-risk features such as BRAF V600E and RAS/TP53 co-mutations. Broader implementation raises ethical challenges related to organ allocation, driving interest in alternative strategies including living-donor LT and innovative procedures such as RAPID. This narrative review summarizes evolving evidence supporting LT for CRLM, current approaches to patient selection, oncological outcomes, and practical considerations for implementing LT into contemporary HPB and transplant practice.

## Simple Summary

Many patients with colorectal cancer develop liver metastases that cannot be removed by surgery. Traditionally, these patients have been treated with systemic therapy, but long-term survival remains limited. Recent studies have shown that liver transplantation can provide excellent survival outcomes in carefully selected patients with cancer confined to the liver. As interest in this treatment grows, there is a need to better understand the evidence supporting its use, how suitable candidates should be selected, and the challenges of implementing transplantation in clinical practice. This review summarizes current knowledge on liver transplantation for unresectable colorectal liver metastases and discusses its potential role in the future management of metastatic colorectal cancer.

## 1. Introduction

Colorectal cancer (CRC) remains a leading cause of cancer-related mortality worldwide [1], and approximately 50% of patients will develop colorectal liver metastases (CRLM) during the course of their disease [2,3,4]. Patients presenting with liver-only or liver-dominant metastatic disease can be candidates for resection or ablation of liver metastases, with 5-year overall survival (OS) rates of 30–55% [5]. Systemic anti-cancer treatment has expanded the population eligible for curative-intent surgery, either by converting technically unresectable disease to resectable disease through downstaging (induction therapy) or by refining patient selection among technically resectable patients through assessment of tumor biology (neoadjuvant therapy) [5]. For the larger cohort with permanently unresectable liver-confined CRLM, prognosis remains poor with palliative chemotherapy, providing a median OS of 24–30 months and 5-year OS below 15% [6,7].

Liver transplantation (LT) for CRLM was explored in the 1980s and 1990s but was largely abandoned because of poor survival (5-year OS < 20%) and ethical concerns regarding allocating scarce donor organs to patients with metastatic disease [8,9]. Over the last two decades, advances in perioperative management, systemic chemotherapy, immunosuppression, and understanding of tumor biology have fundamentally changed this landscape and prompted a re-evaluation of LT as a potentially curative option for a carefully defined subgroup of patients with CRLM [9]. Importantly, LT for CRLM has evolved from an indiscriminate, recurrence-prone rescue strategy to a highly stratified treatment approach guided by clinical risk models, functional imaging, and molecular profiling [10,11,12]. In this context, long-term survival now appears comparable to outcomes achieved with transplantation for hepatocellular carcinoma (HCC), carefully selected perihilar cholangiocarcinoma treated according to the Mayo protocol, and selected neuroendocrine liver metastases, provided that similarly rigorous selection criteria are applied [13,14,15,16,17].

This narrative review summarizes contemporary evidence supporting LT for CRLM, with particular emphasis on patient selection, oncological outcomes, and comparison with established non-transplant strategies. It further addresses practical and ethical challenges related to organ allocation, emerging surgical innovations, and future priorities in the field. The aim is to provide gastrointestinal oncologists, HPB surgeons, and transplant teams with a pragmatic framework for identifying suitable candidates, counselling patients, and integrating LT into multidisciplinary treatment algorithms for advanced CRC.

## 2. Liver Resection for CRLM

Liver resection remains the reference standard and cornerstone of curative-intent treatment for patients with CRLM. The last two decades have seen continuous refinements in surgical technique with parenchymal-sparing approaches, two-stage hepatectomy, and portal vein embolization and double-vein embolization, and adjunctive ablative approaches. Together with modern systemic chemotherapy, these refinements have expanded the limits of resectability while improving perioperative safety and long-term oncological outcomes [5,18]. In carefully selected patients, these advances translate into 5-year OS rates of 30–55% [19,20].

Nevertheless, liver resection remains inherently constrained by tumor burden, anatomical distribution, vascular involvement, and the requirement to preserve an adequate future liver remnant. Consequently, a substantial proportion of patients remain permanently unresectable despite optimal conversion strategies. Furthermore, recurrence following complete resection occurs in about 70%, particularly within the liver, reflecting both residual micrometastatic disease and unfavorable tumor biology [20]. Overall, these limitations define the current boundaries of liver resection as a curative strategy for advanced CRLM.

Within this context, increasing clinical interest has focused on alternative strategies for patients with unresectable, liver-only disease. The feasibility and oncological potential of LT in this setting were first systematically evaluated in the modern era by the SECA trials [21,22].

## 3. The SECA Trials

The modern evidence supporting LT for unresectable CRLM originates from the prospective SECA program conducted at Oslo University Hospital. SECA-I was designed as a proof-of-concept pilot study evaluating the feasibility, safety, and long-term oncological outcomes of LT in patients with unresectable liver-only CRLM following systemic chemotherapy. Apart from the requirement for liver-only disease, the cohort represented a broad clinical spectrum, including patients with markedly different tumor burdens and biological behavior. This heterogeneity proved instrumental in identifying the prognostic factors that subsequently formed the basis of modern selection criteria. Although limited by its single-center, non-randomized design and relatively small sample size, the study represented the first prospective evaluation of LT for this indication and established the foundation for subsequent clinical studies and international collaboration.

In the SECA-I pilot trial, 21 patients with non-resectable, liver-only CRLM who had received at least 6 weeks of chemotherapy underwent LT, yielding an estimated 5-year OS of 60%, comparable to outcomes reported for HCC within Milan criteria [21,22]. Unlike subsequent studies, SECA-I enrolled a heterogeneous patient population, including patients with progressive disease at the time of transplantation. Consequently, outcomes ranged from rapid recurrence and short survival to durable long-term remission exceeding 15 years, illustrating the profound influence of tumor biology. This heterogeneity subsequently enabled identification of the prognostic factors that formed the basis of the Oslo Score and later patient selection criteria. Building on these findings, the subsequent SECA-II trial applied more stringent selection criteria and reported an estimated 5-year OS of 83%, confirming the potential for excellent long-term survival in highly selected patients [22,23].

The SECA trials also defined a characteristic pattern of recurrence following LT. Although disease-free survival (DFS) was relatively short, with a median of approximately 12 months, post-recurrence survival was frequently long. Many patients lived for several years after recurrence, and a substantial proportion underwent metastasis-directed treatments with curative intent, most commonly for lung metastases [24]. In selected cases, complete remission was achieved with survival exceeding 10 years following pulmonary metastasectomy. Post-transplant chemotherapy was generally well-tolerated, and no episodes of graft rejection were observed [25]. Notably, some patients achieved durable responses to systemic regimens that had previously been ineffective before LT. These observations support the hypothesis that total hepatectomy may effectively eliminate the dominant hepatic tumor burden and may alter the biological behavior of residual microscopic disease. Consequently, rigorous post-transplant surveillance and an aggressive metastasis-directed treatment strategy represent an integral component of this therapeutic approach, rather than reliance on palliation management alone [24,26].

Collectively, the Oslo experience provides not only a proof-of-concept that LT can achieve long-term survival in selected patients with unresectable CRLM, but also illustrates how iterative refinement of patient selection transformed an initially heterogeneous pilot experience into a reproducible treatment strategy supported by prospective studies and randomized evidence.

## 4. Immunosuppression

In the SECA program, immunosuppression consisted of corticosteroids, mycophenolate mofetil, and sirolimus, an mTOR inhibitor. In the early SECA experience, sirolimus was initiated immediately after transplantation and targeted at higher trough concentrations than those currently recommended. However, this strategy was associated with impaired wound healing and a high incidence of incisional hernias. Consequently, subsequent protocols delayed introduction of mTOR inhibition until 4–6 weeks after transplantation and adopted lower target trough concentrations (5–10 ng/mL), consistent with contemporary international recommendations. Corticosteroids were gradually tapered and discontinued within six months after LT.

To date, no high-quality disease-specific evidence supports one immunosuppressive regimen over another in patients undergoing LT for CRLM, and current practice is largely extrapolated from HCC. In HCC, mTOR inhibitors combined with reduced-exposure calcineurin inhibitors have been associated with lower recurrence rates and improved OS following LT [27,28]. Current international consensus recommendations therefore advocate minimization of calcineurin inhibitor exposure with introduction of an mTOR inhibitor or delayed conversion to an mTOR-based regimen approximately 4–6 weeks after transplantation to reduce wound-healing complications while potentially preserving antitumor efficacy [29].

Although these data provide a rationale for mTOR-containing regimens in patients transplanted for CRLM, the evidence remains indirect, and no immunosuppressive strategy has yet been prospectively validated for this indication. Importantly, although chronic immunosuppression is associated with an increased incidence of several de novo malignancies following solid organ transplantation, particularly cutaneous squamous cell carcinoma, basal cell carcinoma, and post-transplant lymphoproliferative disorders, current clinical experience does not suggest that it accelerates recurrence or fundamentally alters the biological behavior of CRLM in carefully selected patients. Most recurrences after LT for CRLM occur as isolated pulmonary metastases and demonstrate relatively slow growth, suggesting that tumor biology, rather than immunosuppression, remains the principal determinant of long-term outcome [30].

## 5. Post-Transplant Recurrence: Patterns, Surveillance and Management

Recurrence after LT for CRLM is common but typically follows a favorable clinical course. An analysis of all 56 patients transplanted within the SECA program between 2006 and 2019 demonstrated recurrence in 44 patients (79%) [26]. The lung was the first site of recurrence in more than half of patients, followed by the liver, lymph nodes, and other sites. Median disease-free survival was approximately 9 months, and nearly all recurrences occurred within the first two years after transplantation. However, many patients remained candidates for metastasis-directed therapy and experienced prolonged survival after recurrence.

Management of recurrence is fundamentally different from conventional palliative treatment strategies. All patients with recurrent disease in the SECA program received active treatment, with approximately half initially undergoing surgery, ablation, or chemoradiotherapy with curative intent. Pulmonary metastasectomy was particularly effective, achieving a 5-year overall survival approaching 70% from the time of lung resection, whereas curative-intent treatment of recurrence at other sites resulted in lower long-term survival. Most patients subsequently received systemic chemotherapy, which appeared feasible in combination with immunosuppressive therapy, although gastrointestinal and dermatological toxicities may be more frequent, particularly with irinotecan- and EGFR inhibitor-based regimens [25].

Given the high incidence but frequently indolent and potentially treatable nature of recurrence, structured long-term surveillance is an integral component of the transplant strategy rather than simply postoperative follow-up. Within the Oslo protocols, patients are reviewed monthly during the first postoperative year, every three months during the second year, and every six months thereafter. Contrast-enhanced CT is performed every three months during the first two years and every six months thereafter in recurrence-free patients. This intensive surveillance, combined with multidisciplinary assessment involving transplant, HPB, thoracic surgery and oncology teams, facilitates early identification of limited recurrences, most commonly slowly growing pulmonary metastases, that remain amenable to curative-intent treatment.

## 6. Refining Patient Selection: Prognostic Factors and Scoring Systems

The survival outcomes observed in the Norwegian studies are intrinsically linked to the rigorous selection of patients with favorable tumor biology. The initial SECA-I trial identified several negative prognostic factors, which were subsequently incorporated into the Oslo Score [31]. In parallel, metabolic tumor volume measured by ^18^F-FDG PET/CT (PET-MTV) has emerged as a robust indicator of total viable tumor burden [32]. In patients undergoing LT for CRLM, low PET-MTV (<70 cm^3^) has been consistently associated with improved OS and a lower risk of recurrence, independent of conventional size- and number-based criteria [11]. In clinical practice, PET-MTV may help distinguish biologically indolent disease from diffuse, highly metabolically active metastases in patients who may otherwise appear similar on standard cross-sectional imaging. Accordingly, PET-MTV < 70 cm^3^ has now been incorporated into pretransplant selection algorithms at Oslo University Hospital alongside the Oslo Score (Table 1).

The prognostic value of the Oslo Score is substantial. Among the 60 patients transplanted for CRLM at Oslo University Hospital between 2006 and 2023, patients with an Oslo Score of 0 or 1 achieved 10-year overall survival (OS) rates of 88.9% and 80.0%, respectively, whereas patients with scores of 3 or 4 had a median OS of only 24.8 months and a 5-year OS of 8.3% [14]. From a pragmatic clinical perspective, LT should generally be restricted to patients with an Oslo Score of 0–2 and disease controlled on systemic therapy, whereas higher scores should be regarded as markers of unfavorable tumor biology. Additional high-risk features, including right-sided primary tumors and pN2 nodal involvement, may favor alternative locoregional or systemic treatment strategies.

Beyond clinical and radiological criteria, molecular prognostic factors are increasingly important in transplant oncology. The IHPBA consensus guidelines recommend that pN2 nodal involvement of the primary tumor should generally be considered a relative contraindication to LT [29]. This recommendation is supported by the SECA-II Arm D study, which specifically evaluated extended recipient criteria, including patients with advanced primary tumors (pN2 disease) [33]. Patients with pN2 disease demonstrated significantly inferior survival outcomes compared to those with node-negative or pN1 disease, suggesting pN2 status as a robust negative prognostic factor. These findings have been corroborated in larger institutional cohorts, in which even patients with favorable Oslo scores (0–1) had poor outcomes in the presence of pN2 disease. These findings have subsequently informed institutional selection strategies and current consensus recommendations. Consequently, pN2 disease has been adopted as an exclusion criterion in subsequent Oslo protocols.

Recent molecular studies have further refined these criteria. Moosavi et al. demonstrated that combined *RAS* and *TP53* mutations were associated with a significantly inferior survival after LT, with a median OS of 21 months compared with 60 months in patients without these co-mutations [12]. Importantly, the prognostic impact of these molecular alterations appeared more pronounced after LT than after liver resection, highlighting the importance of molecular profiling in personalized transplant decision-making. Consequently, comprehensive molecular profiling, including *RAS*, *BRAF*, *TP53,* and mismatch-repair status, should routinely be incorporated into candidate evaluation whenever available before listing a patient for LT. Unfavorable molecular profiles, particularly *RAS*/*TP53* co-mutations and *BRAF* V600E mutations, should generally be regarded as contraindications to transplantation.

## 7. Clinical Trial Evidence: LT vs. Standard of Care

The strongest evidence supporting LT for CRLM comes from the TransMet trial, the first multicenter, open-label, prospective, randomized controlled trial comparing LT plus chemotherapy with chemotherapy alone in patients with permanently unresectable CRLM [16]. The trial enrolled highly selected patients with *BRAF* wild-type CRC, absence of extrahepatic disease, and disease controlled on systemic chemotherapy. The primary endpoint was 5-year OS. The results demonstrated a substantial survival advantage: 5-year OS was 56.6% in the LT plus chemotherapy group compared with 12.6% in the chemotherapy-alone group (hazard ratio [HR] 0.37; 95% confidence interval [CI] 0.21–0.65; *p* = 0.0003) in the intention-to-treat analysis, as summarized in Table 2. Per-protocol analysis demonstrated an even greater survival advantage, with a 5-year OS of 73.3% in the LT group. Although TransMet provides the highest level of evidence currently available, interpretation should acknowledge that enrolled patients represented a highly selected subgroup with chemotherapy-responsive, liver-only, BRAF wild-type disease managed at experienced transplant centers. Consequently, although the trial strongly supports LT in appropriately selected patients, the findings should not automatically be extrapolated to the broader metastatic colorectal cancer population. Taken together, these findings provide the highest level of evidence to date that LT, when combined with systemic chemotherapy and rigorous patient selection, offers a substantial survival benefit over the current standard of care (palliative chemotherapy) for this patient population.

Importantly, no other systemic or locoregional treatment strategy in metastatic gastrointestinal cancer has achieved survival outcomes comparable to those observed with LT in highly selected patients with unresectable, liver-only CRLM. Taken together, the SECA-I and SECA-II proof-of-concept studies and the randomized TransMet trial demonstrate that LT can achieve long-term survival far beyond what is currently attainable with chemotherapy, immunotherapy, or targeted therapy alone in microsatellite-stable disease, thereby establishing LT as a central evidence-based strategy within modern transplant oncology for this patient population.

Nevertheless, current selection criteria restrict LT eligibility to only a very small subset of patients with metastatic CRC. An analysis of the first-line NORDIC-VII trial population illustrates this challenge: among 566 patients initiating palliative chemotherapy, only 47 patients (8%) had liver-only disease, Eastern Cooperative Oncology Group (ECOG) performance status 0–1, and age ≤ 65 years [34]. Application of additional exclusion criteria, including right-sided or ascending colon primary tumors, (y)pN2 nodal involvement, RAS/TP53 co-mutations, PET-MTV > 70 cm^3^, largest CRLM > 5.5 cm, and plasma CEA > 80 ng/mL, would likely reduce the eligible proportion to only 1–3% of patients starting palliative chemotherapy. This is consistent with the experience at Oslo University Hospital, where only 25 patients were enrolled in the ongoing SECA-II program over approximately 11 years in a population of ~5.5 million, corresponding to approximately two transplants per year. In Norway, where approximately 100 liver transplantations are performed annually, the addition of two patients per year with CRLM to the waiting list is unlikely to materially affect waiting-list times or waiting-list mortality.

## 8. Health-Related Quality of Life and Cost-Effectiveness

Beyond survival, LT for CRLM appears compatible with durable health-related quality of life (HRQOL). In the SECA cohort, baseline HRQOL scores were comparable to those of patients initiating first-line chemotherapy in the NORDIC-VII first-line chemotherapy trial and to an age- and sex-matched Norwegian reference population [35]. Following a transient decline 3 months after LT, global health status and physical functioning returned to baseline by 6 months and remained stable for up to 3 years, despite the fact that most patients developed recurrence and subsequently underwent additional oncologic or surgical treatment. Importantly, baseline symptom burden also appeared prognostic: appetite loss and pain at the time of transplantation were associated with significantly shorter overall survival, suggesting that HRQOL measures may provide clinically relevant information beyond conventional radiologic and oncologic selection criteria [35].

From a health-economic perspective, Norwegian Markov modeling suggests that LT is likely to be cost-effective compared with last-line systemic therapies, including trifluridine/tipiracil (TAS-102) and regorafenib, or best supportive care in patients who have failed second- or third-line chemotherapy [36]. Although LT is associated with substantially higher upfront costs, incremental cost-effectiveness ratios remained within accepted national willingness-to-pay thresholds. These findings support LT for highly selected patients not only as a life-prolonging intervention, but also as a potentially justified use of healthcare resources in healthcare systems with established transplant programs and strategies to mitigate graft scarcity.

## 9. Organ Allocation and Alternative Strategies

The success of LT for CRLM introduces important ethical and logistical challenges, particularly in regions with severe deceased-donor organ scarcity, such as the United States [37]. The IHPBA consensus guidelines explicitly address these concerns, emphasizing the need to consider the broader societal implications of LT for CRLM in organ allocation and management of national transplant waiting lists [29].

Beyond organ scarcity, implementation of LT for CRLM raises broader ethical questions concerning distributive justice and equitable allocation of deceased-donor organs. The balance between maximizing individual patient benefit and preserving fair access for patients with established transplant indications differs substantially across healthcare systems because donor availability, waiting-list mortality, and allocation policies vary considerably between countries. Consequently, implementation should occur within transparent institutional protocols and national allocation frameworks while continuing to evaluate outcomes prospectively. Increased use of living donor transplantation, extended-criteria donor grafts, split-liver transplantation, and RAPID procedures may help expand transplant capacity without substantially affecting access for other transplant candidates.

To circumvent the reliance on the deceased-donor pool, alternative strategies have been explored, most notably Living Donor Liver Transplantation (LDLT). The North American experience, including reports from the Cleveland Clinic and the University of Rochester, has demonstrated the feasibility and success of LDLT for CRLM, with 1.5-year OS rates as high as 100% [38]. A comprehensive U.S. analysis further demonstrated that 56.5% of transplantations performed for CRLM had living donors, underscoring LDLT as a key strategy for overcoming the ethical and logistical limitations associated with deceased-donor allocation [21]. In the same cohort, 3-year overall survival was higher after LDLT than after deceased-donor liver transplantation (DDLT) (71.4% vs. 51.4%), although these findings may partly reflect differences in patient selection and graft quality [37].

Additional approaches to expand transplant availability include the use of extended-criteria donors and novel surgical strategies. One such approach is the RAPID procedure (Resection and partial liver segment II/III transplantation with delayed total hepatectomy), which combines staged hepatectomy with transplantation of a partial graft [39]. RAPID has been proposed as a strategy to increase graft utilization by enabling use of split deceased-donor grafts or partial living-donor grafts. Although still considered experimental, RAPID may be particularly attractive in regions with limited access to full-size grafts and may further facilitate implementation of living-donor programs [40].

A Norwegian survey involving relatives of patients with advanced CRC, LT recipients, and healthcare professionals demonstrated a remarkably high willingness to donate part of the liver to a next of kin, supporting the feasibility of a living-donor program in Norway [41]. Accordingly, a living donor RAPID (LD-RAPID) protocol, combining living donor partial liver transplantation with the RAPID concept, is currently under development at Oslo University Hospital. Importantly, implementation of such programs requires close collaboration among gastrointestinal oncologists, transplant surgeons, and HPB surgeons, and should currently remain confined to experienced transplant oncology centers with established expertise in liver transplantation, complex HPB surgery, transplant hepatology, thoracic metastasectomy, dedicated transplant radiology and pathology, and multidisciplinary decision-making. Although no minimum transplant volume has been defined, implementation should occur within prospective institutional protocols or clinical trials in accordance with current IHPBA recommendations [29].

In Scandinavia, organ allocation is coordinated through Scandiatransplant, and Norway has established a leading LT program for CRLM. As additional Nordic centers develop referral pathways and institutional protocols, regional collaboration and prospective outcome registries will be essential to ensure equitable access and maintain high-quality outcomes across the region.

## 10. Liver Transplant for High Tumor Burden, Resectable Disease

Liver transplantation for technically resectable CRLM remains controversial. However, emerging evidence suggests that LT may provide a survival advantage in carefully selected patients with extensive tumor burden. Dueland et al. compared outcomes after portal vein embolization followed by liver resection with outcomes after LT in patients with advanced CRLM and demonstrated substantially improved 5-year overall survival (OS) among patients with high tumor load (defined as ≥9 lesions or largest lesion ≥5.5 cm) treated with LT compared with resection (33.4% vs. 6.7%) [42]. This benefit appeared particularly pronounced in patients with left-sided primary tumors (45.3% vs. 12.5%). Similarly, Lanari et al. directly compared liver resection and LT using SECA-I selection criteria and reported that, among patients with high tumor burden scores (≥9), long-term survival favored LT over resection (5-year OS 52.2% vs. 22.7%) [43]. Notably, the survival difference reached statistical significance in the subgroup with low Oslo Scores and high tumor burden, in whom 5-year OS was 69.1% after LT compared with 14.6% after resection (*p* = 0.002).

Additional evidence from Oslo University Hospital further supports the potential role of LT in patients with recurrent liver-limited disease after previous hepatectomy. In a recent cohort study comparing repeat liver resection with LT in patients who fulfilled institutional LT criteria, LT was associated with markedly superior long-term survival despite the LT group being considered technically unresectable by multidisciplinary assessment [44]. Patients undergoing LT achieved a 5-year OS of 90% compared with 44% after repeat liver resection, while disease-free survival was similar between groups. Importantly, survival after recurrence was substantially longer following LT (median 116.6 vs. 26.1 months), and recurrences after LT were exclusively pulmonary and frequently amenable to curative-intent lung resection. These findings further support the concept that total hepatectomy may fundamentally alter recurrence patterns and improve long-term disease control in selected patients with advanced CRLM.

Hepatic artery infusion (HAI) therapy has emerged as an important liver-directed treatment strategy for selected patients with unresectable CRLM and has been proposed as a potential alternative to LT in some highly selected patients [45,46,47]. HAI can be delivered either using subcutaneous pumps with floxuridine (FUDR) or using direct catheter injection with oxaliplatin. HAI, especially with FUDR, allows extremely high concentration of chemotherapy in the liver, and proponents suggest that its effects resemble that of LT. A recent single-center analysis from Memorial Sloan Kettering Cancer Center, where HAI pump treatment was pioneered, suggested that survival after HAI pump treatment in transplantable patients can be similar to that of liver transplant [47]. A French randomized trial reported promising results of adjuvant HAI oxaliplatin after local treatment of liver metastases [48], while a Dutch randomized trial reported no improvement of progression-free survival or OS after adjuvant HAI pump treatment with FUDR [49]. However, comparisons between HAI and LT should be interpreted cautiously because published studies include fundamentally different patient populations, indications, and selection criteria. At present, no prospective study has directly compared these treatment strategies. A Norwegian phase 2 randomized trial has explored the effect of HAI pump with FUDR in unresectable CRLM (NCT04898504), with results pending later this year, while a phase 3 randomized trial is ongoing in the USA (NCT05863195). These studies will hopefully provide evidence to the role of HAI treatment in CRLM, including its role in patients eligible for LT.

These findings suggest that LT may offer superior long-term oncologic outcomes compared with even advanced resection strategies in selected patients with technically resectable but biologically aggressive disease, particularly in those with left-sided primary tumors, low Oslo Scores, and high tumor burden. These observations further support the hypothesis that total hepatectomy may eliminate residual microscopic intrahepatic disease that contributes to recurrence following conventional resection. Nevertheless, the available evidence remains limited to retrospective comparisons and single-center experiences. Prospective validation through randomized trials will therefore be essential before LT can be recommended over liver resection in biologically high-risk but technically resectable patient population.

## 11. Conclusions

LT for permanently unresectable, liver-only CRLM can achieve long-term survival comparable to transplantation for selected primary liver malignancies in carefully selected patients. Contemporary practice should emphasize strict selection based on chemotherapy-responsive liver-only disease, validated clinical and molecular risk stratification, and treatment within experienced centers operating under formal protocols or prospective clinical trials. Future studies should further clarify the role of LT in biologically high-risk disease and in patients with technically resectable but high-burden CRLM.

Despite the promising results, several limitations remain. Most available data derive from single-center experiences with relatively small patient cohorts, and the randomized TransMet trial included a highly selected population with liver-only, BRAF wild-type, chemotherapy-responsive disease. Consequently, the benefit of LT in patients with unfavorable molecular profiles or right-sided primary tumors remains uncertain. Future research should focus on molecular risk stratification, circulating tumor DNA (ctDNA)-guided selection and surveillance [50], immune profiling, radiomic biomarkers, and artificial intelligence-assisted prediction models. Prospective international registries and randomized trials will be essential to further refine patient selection, optimize immunosuppressive strategies, and define the role of LT alongside emerging liver-directed therapies.

## Figures and Tables

**Table 1:** Clinical prognostic scoring systems used for patient selection before liver transplantation for unresectable colorectal liver metastases. Higher Oslo Scores indicate less favorable tumor biology and poorer long-term survival. PET-MTV = metabolic tumor volume measured on ^18^F-FDG PET/CT. OS = overall survival.

Prognostic Scoring System	Components (1 Point Each)	Reported Prognostic Value	Source
**Oslo Score**	1. Largest tumor size ≥ 5.5 cm	5-year OS: 63.4% (Score 0–2) vs. 8.3% (Score 3–4) [1]	Dueland 2020 [31]
	2. Progressive disease while on chemotherapy		
	3. Plasma CEA level > 80 µg/L		
	4. Time from primary tumor resection to LT < 2 years		
**Metabolic Tumor Volume (PET-MTV)**	Measured by ^18^F-FDG PET/CT	PET-MTV < 70 cm^3^ associated with improved OS [6]	Grut 2022 [32]

**Table 2:** Five-year overall survival in the randomized TransMet trial comparing liver transplantation plus standard systemic chemotherapy with systemic chemotherapy alone in patients with permanently unresectable colorectal liver metastases.

Treatment Group	5-Year Overall Survival (Intention-to-Treat)	Hazard Ratio (HR)	*p*-Value
**LT plus Chemotherapy**	56.6% (95% CI 43.2–74.1)	0.37 (95% CI 0.21–0.65)	0.0003
**Chemotherapy Alone**	12.6% (95% CI 5.2–30.1)		

LT = liver transplantation; HR = hazard ratio; CI = confidence interval. Patients received contemporary guideline-based systemic chemotherapy according to the TransMet protocol.

## Data Availability

No new data were created or analyzed in this study. Data sharing is not applicable to this article.
